# [Corrigendum] Antitumour activity of TH1579, a novel MTH1 inhibitor, against castration-resistant prostate cancer

**DOI:** 10.3892/ol.2026.15853

**Published:** 2026-09-10

**Authors:** Mingqiu Hu, Jing Ning, Likai Mao, Yuanyuan Yu, Yu Wu

Oncol Lett 21: 62, 2021; DOI: 10.3892/ol.2020.12324

Following the publication of the above paper, an interested reader drew the Editor's attention to the fact that, regarding the western blots shown in Fig. 2C on p. 5, the β-actin blots shown for the PC-3 cell line were very similar in appearance to the blots shown for the H2AX protein for the DU-145 cell line, albeit with some vertical resizing of the bands in the latter case.

The authors have re-examined their data, and realize that the western blots in Fig. 2C were inadvertently assembled incorrectly. The revised version of Fig. 2, now showing the correct blots for the H2AX protein for the DU-145 cell line in Fig. 2C, is shown on the next page. The authors are grateful to the Editor of *Oncology Letters* for allowing them this opportunity to publish a Corrigendum, and all the authors agree with its publication. They also apologize to the readership for any inconvenience caused.

## Figures and Tables

**Figure:**
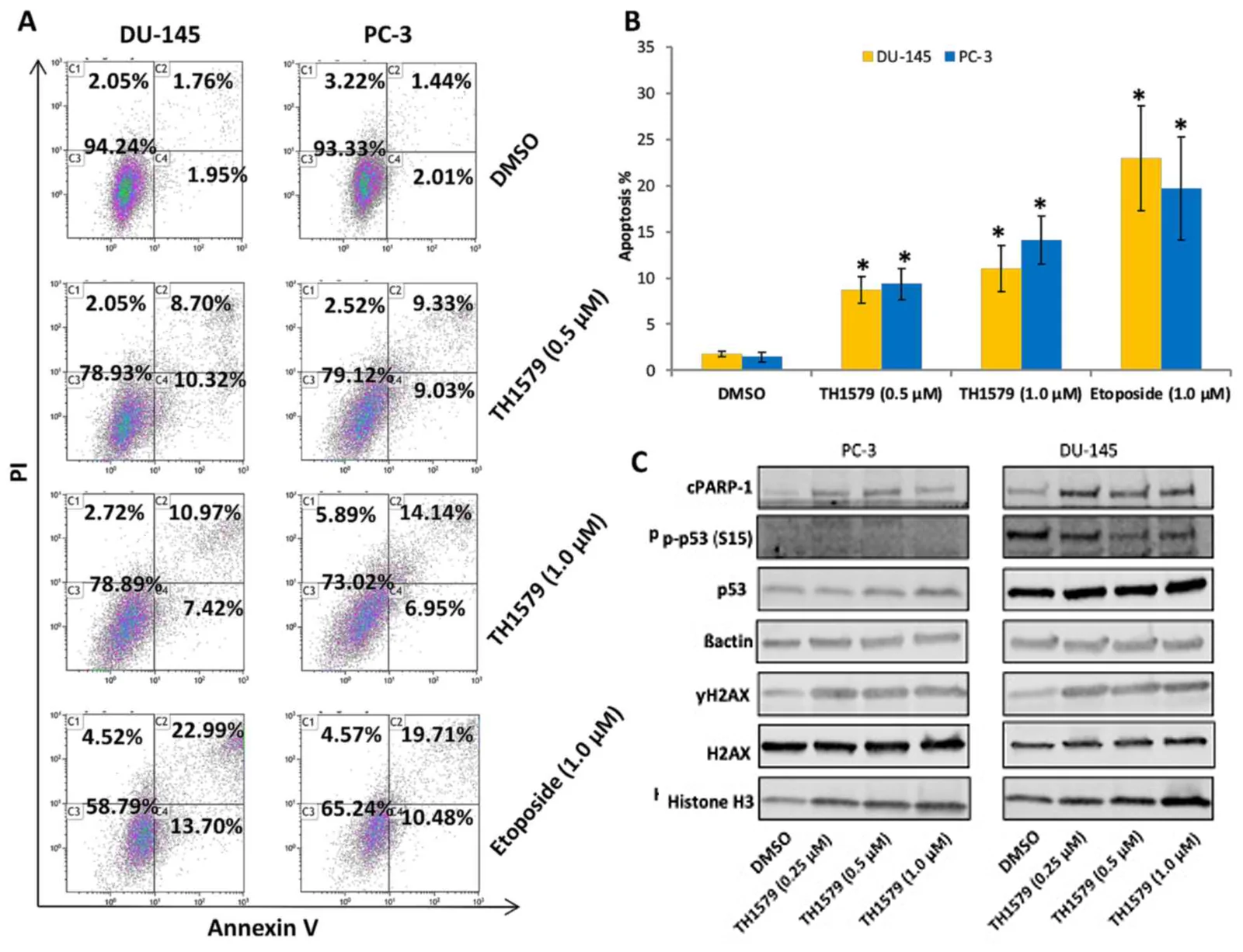
TH1579 induces apoptosis in castration-resistant prostate cancer. (A) Representative images of flow cytometric analysis highlighting apoptosis in DU-145 and PC-3 cells treated with TH1579 for 72 h. (B) Flow cytometric quantification of apoptosis in PC-3 or DU-145 cells following PI and Annexin V staining. Values represent the mean ± SEM from three independent experiments. *P<0.01, each experiment group [including TH1579 (1.0 µM), TH1579 (0.5 µM) or Etoposide group vs. DMSO group]; Tukey's test. (C) Representative western blot images of cPARP-1, p-p53 (S15) and γH2A.X expression levels from >3 independent experiments 24 h after incubation with TH1579. cPARP-1, cleaved poly (ADP-ribose) polymerase 1; PI, propidium iodide..

